# High-fidelity 3D models of human cadavers and their organs with the use of handheld scanner–Alternative method in medical education and clinical practice

**DOI:** 10.3389/fmed.2025.1644808

**Published:** 2025-09-24

**Authors:** Weronika Michalik, Magdalena Szczepanik, Konrad Biel, Michał Mordarski, Kacper Bak, Kamila Fryzlewicz, Karol Jaszewski, Szymon Maciaszek, Monika Pierzchała, Areej Arshad, Daniel Rams, Jerzy Walocha, Halina Dobrzynski, Małgorzata Mazur

**Affiliations:** ^1^Department of Anatomy, Jagiellonian University Medical College, Kraków, Poland; ^2^Centre for Digital Medicine and Robotics - Jagiellonian University Medical College, Kraków, Poland; ^3^Medical Informatics Engineering, Fort Wayne, IN, United States; ^4^University of Manchester, Manchester, United Kingdom

**Keywords:** human anatomy, 3D scanning, cadaveric dissection, new technologies in medicine, anatomical education

## Abstract

**Background:**

Traditional cadaveric dissection is considered the gold standard in anatomical education; however, its accessibility is limited by ethical, logistical, and financial constraints. Recent advancements in three-dimensional (3D) scanning technologies provide an alternative approach that enhances anatomical visualization while preserving the fidelity of real human specimens.

**Aim:**

This study aimed to create digitized 3D models of dissected human cadaveric specimens using a handheld structured-light scanner, thus providing a sustainable and accessible resource for educational and clinical applications.

**Methods:**

Eight human cadaveric specimens were dissected and scanned using the Artec 3D Spider handheld scanner. The obtained scans were processed in Artec Studio 17 Professional and further processed in Blender software. Finalized 3D models were exported in.MP4 format and paired with two-dimensional (2D) images for enhanced anatomical understanding.

**Results:**

A total of 12 anatomical 3D models were successfully created, capturing detailed anatomical landmarks with a resolution of 0.1 mm and an accuracy of 0.05 mm. The models encompassed key anatomical regions or organs, including the brain, skull, face, neck, thorax, heart, abdomen, pelvis, and lower limb. The combination of 3D models alongside 2D images allowed for interactive and immersive learning, as well as improving spatial comprehension of complex anatomical structures.

**Conclusion:**

The use of high-fidelity 3D scanning technology provides a promising alternative to traditional dissection by offering an accessible, sustainable, and detailed representation of spatial relationships in the human body. This approach enhances medical education and clinical practice, bridging the gap between theoretical knowledge and practical application.

## 1 Introduction

The detailed anatomical knowledge is one of the most significant factors in clinical excellence regardless of medical specialty (1). Medical trainees as well as experienced clinicians are challenged by the complexity of human body structure and often return to anatomical foundations, recall key structures with their locations and refine surgical techniques and approaches (2).

Dissecting human cadavers has been recognized as the most effective method to understand anatomical structures, spatial relationships and the individuality of each human organism (1, 3–5). However, anatomical dissection facilities face several technical limitations, such as low availability and evanescence of human specimens, plus resource-intensive and carcinogenic preservation technique (1) along with jurisdictional differences in regulatory policies (6).

Recent technological advancements have proven that virtual models are an effective resource in anatomical studies that can overcome many constraints inherent to cadaveric method (2, 7–9). Three-dimensional (3D) visualization technology has introduced “new medicine,” enhancing medical practices, being used for surgical planning and education of trainees and patients (10, 11). Previous studies have shown that 3D models can supplement traditional teaching methods and are superior to two-dimensional (2D) imaging in teaching complex anatomy (7, 9). Among many methods of creating 3D scans (12), the handheld, structured-light 3D scanner acquires precise details of the surface characteristics of an object by directing the light source at the object and scanning the desired target, in a quick and convenient workflow without any special lightning. Finally, the result of the 3D model converted from series of 2D images, can be displayed and manipulated remotely by the viewers.

Given the demanding nature of clinical schedules and varying levels of accessibility and familiarity with new technologies, there is a growing need for an educational solution that maximizes teaching efficiency and allows for integration into daily clinical practice. As anatomical knowledge is universal, it should be readily accessible, in the most reliable form based on human cadaveric specimens.

Considering the potential use of 3D models in anatomical studies and education (7, 12), the aim of this study was to make 3D models of dissected human cadaveric specimens and highlight their main anatomical landmarks. 2D images and 3D models were created to provide interactive and immersive learning content for students, researchers, and clinicians, offering an opportunity to gain a deeper understanding of the structure and function of the human body. Ultimately, this research aimed to provide a promising, sustainable solution to combine cadaver-based anatomy with 3D imaging technology.

## 2 Materials and methods

### 2.1 Ethical consideration and specimen preparation

The research was approved by the Institutional Review Board of the Jagiellonian University (no. 118.0043.1.269.2024). All human cadavers (*n* = 8) used in this study, were provided by an educational body donation program at the Department of Anatomy, Jagiellonian University Medical College in Cracow, Poland. All the cadavers included in the program were obtained up to 72 h after death and are proven negative for COVID-19. All specimens specifying the form of dissection as well as the sex, age, and ethnicity of the deceased are listed in Table 1. Firstly, all cadavers used in this study were perfused with a 10% formalin (36% formaldehyde in methanol) aqueous solution via the femoral artery, then stored in containers filled with the 25% formalin solution for 2 years, then dissected. Only cadavers without any macroscopically visible malformations or pathologies were selected for dissection. The specimens were dissected using surgical and microsurgical instruments including scalpels, forceps, scissors and tweezers. Until the scanning process, all the isolated specimens were kept in boxes filled with 6% formalin solution, whereas the whole cadaveric dissected bodies were covered in 11% formalin solution.

**Table 1:** List of human cadaveric specimens used in the study for the scanning process, highlighting the form of dissection and the sex of the body.

**Cadaver specimen number**	**Sex**	**Age (years)**	**Ethnic group**	**Form of dissection**
1	Male	55	West Slavic	Isolated specimen
2	Male	48	West Slavic	Whole cadaveric body
3	Male	68	West Slavic	Whole cadaveric body
4	Female	72	West Slavic	Whole cadaveric body
5	Female	74	West Slavic	Isolated specimen
6	Male	70	West Slavic	Whole cadaveric body
7	Male	57	West Slavic	Isolated specimen
8	Female	69	West Slavic	Whole cadaveric body

List of human cadaveric specimens used in the study, highlighting the form of dissection, sex, age and ethnicity of the deceased.

The final number of cadavers included in this study was determined by choosing those cadavers in which the key anatomical region was best preserved and intact.

### 2.2 3D scanning and model production

Using an industrial, hand-held scanner “Artec 3D Spider” based on blue-light technology and a licensed software “Artec Studio 17 Professional” installed onto a notebook, every specimen was appropriately scanned repetitively to capture key anatomical structures. All key structures were clearly visualized with 0.1 mm resolution and 0.05 mm accuracy of details. Next, all obtained scans from one specimen were aligned to create a 3D model in “Artec Studio 17 Professional.” The models were exported to meshes then opened and edited to a final version in a free software—Blender [Blender 4.2.3.LTS].

The final versions of animated 3D models were saved in .MP4 format. Based on the obtained videos, keyframes were captured, offering views of the most essential anatomical structures of every specimen, and appropriate descriptions using valid anatomical nomenclature in English were labeled.

## 3 Results

The 3D models were generated with a resolution of 0.1 mm and an accuracy of 0.05 mm, ensuring a high level of anatomical detail. Based on eight human cadaveric specimens described in Table 1, a total of 12 3D models were successfully created and documented in the .MP4 format (Supplementary Videos S1–11), each corresponding to specific anatomical regions. The models provided a detailed representation of key anatomical landmarks, ensuring a high level of accuracy and visualization fidelity.

The 3D models were paired with 2D images (Figures 1–11) to facilitate interactive and comprehensive anatomical understanding. To avoid repetition, each figure is described in detail in its corresponding figure legend.

**Figure 1:**
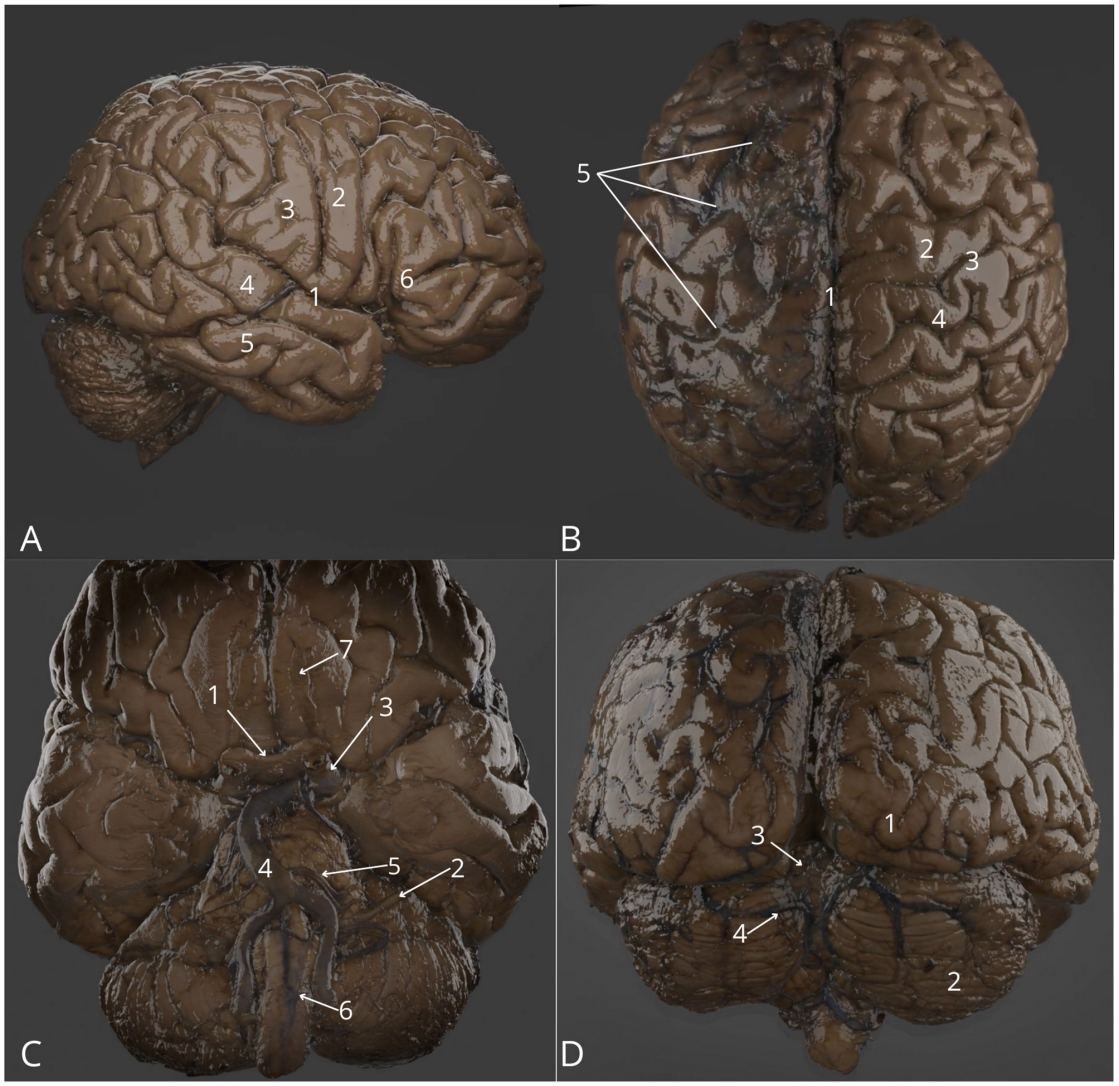
Cerebrum and spinal cord. **(A)** Lateral view: right lateral sulcus (1), right precentral gyrus (2), right postcentral gyrus (3), right superior temporal gyrus (4), right middle temporal gyrus (5), right inferior frontal gyrus–triangular part (6); **(B)** Both hemispheres: superior view with intact arachnoid mater on the left hemisphere focused on longitudinal cerebral fissure (1), precentral gyrus (2), central sulcus (3), postcentral sulcus (4), arachnoid mater (5); **(C)** Both hemispheres: inferior view, brainstem and cerebellum with vascularization, focus on optic chiasm (1), left facial and vestibulocochlear nerves (2), left internal carotid artery (3), basilar artery (4), left anterior inferior cerebellar artery (5), anterior spinal artery (6), left olfactory bulb (7); **(D)** Cerebral hemispheres: posterior view with brainstem and cerebellum, highlighting right occipital pole (1), right hemisphere of cerebellum (2), vermis of cerebellum (3), left superior cerebellar artery (4). The 3D model of this specimen is available in the supplementary material as Supplementary Video S1.

**Figure 2:**
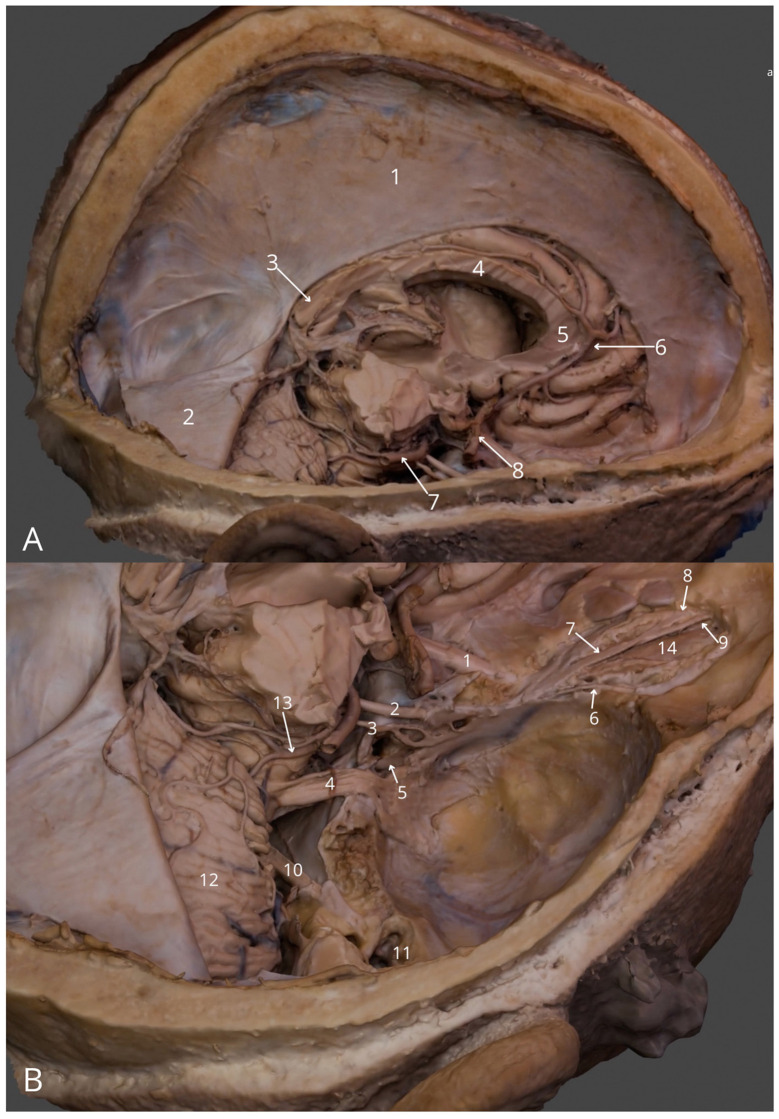
Intracranial structures: **(A)** general view (right): falx cerebri (1), right half of the tentorium cerebelli (2), splenium of corpus callosum (3), body of corpus callosum (4), genu of corpus callosum (5), right anterior cerebral artery (6), right posterior cerebral artery (7), cavernous part of right internal carotid artery (8); **(B)** superior posterior view into the cranial fossae: right optic nerve (1), right oculomotor nerve (2), right trochlear nerve (3), right trigeminal nerve (4), right abducens nerve (5), right lacrimal nerve (6), right frontal nerve (7), right supratrochlear nerve (8), right supraorbital nerve (9), right facial and vestibulocochlear nerves (10), right tympanic cavity (11), right cerebellar hemisphere (12), right superior cerebellar artery (13), right levator palpebrae superioris muscle (14). The 3D model of this specimen is available in the supplementary material as Supplementary Video S2.

**Figure 3:**
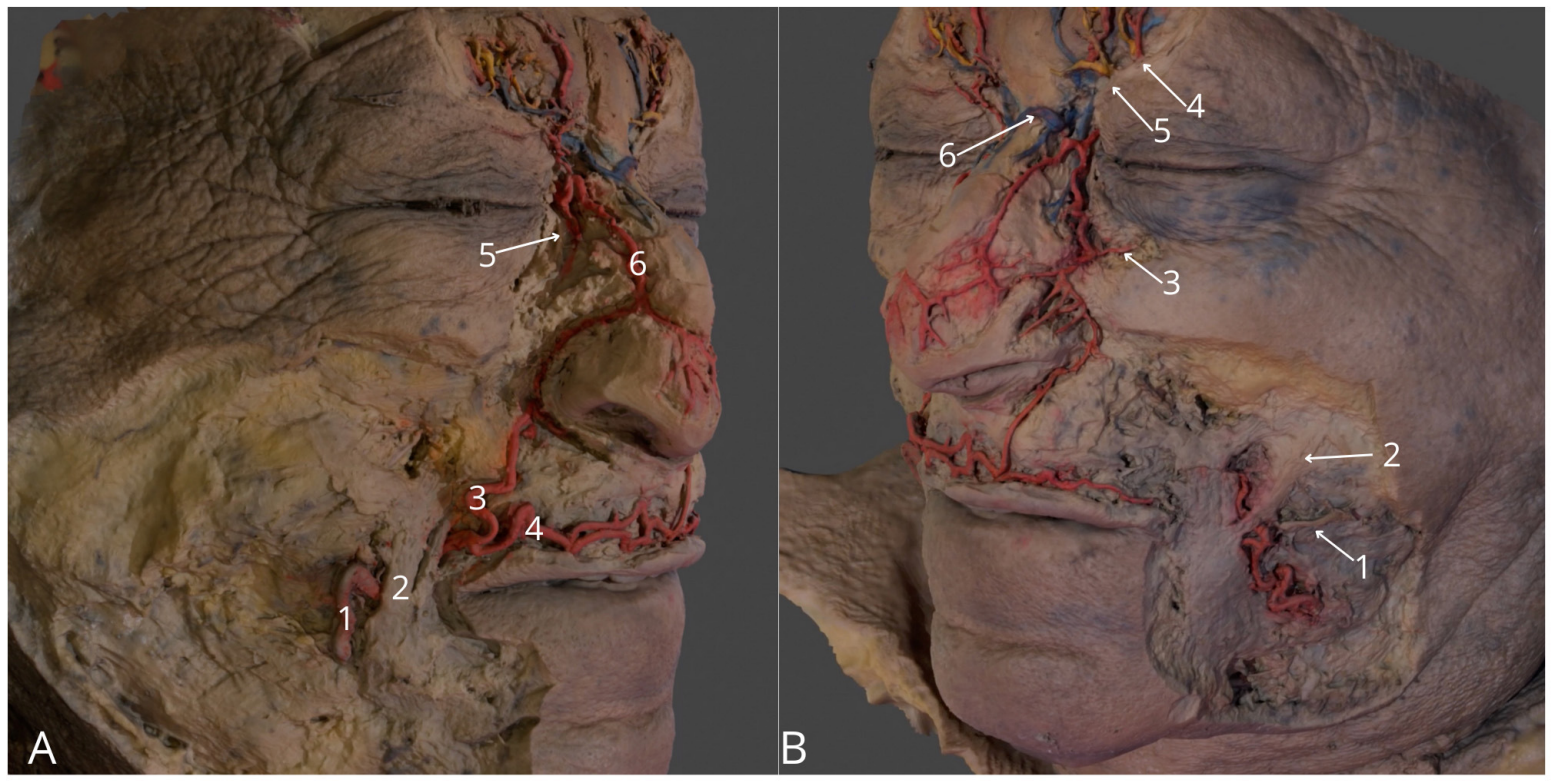
Region of the face. **(A)** Right side, highlighting facial artery (1), right angular artery (3), right superior labial artery (4), anastomosis between right angular artery and right dorsal nasal artery (5), external nasal branch of the right dorsal nasal artery (6); **(B)** Left side, highlighting left parotid duct (1), left zygomatic major muscle (2), left medial palpebral artery (3), left supraorbital vessels with left supraorbital nerve (4), left supratrochlear vessels with left supratrochlear nerve (4) anastomosis between supratrochlear veins (6). The 3D model of this specimen is available in the supplementary material as Supplementary Video S3.

**Figure 4:**
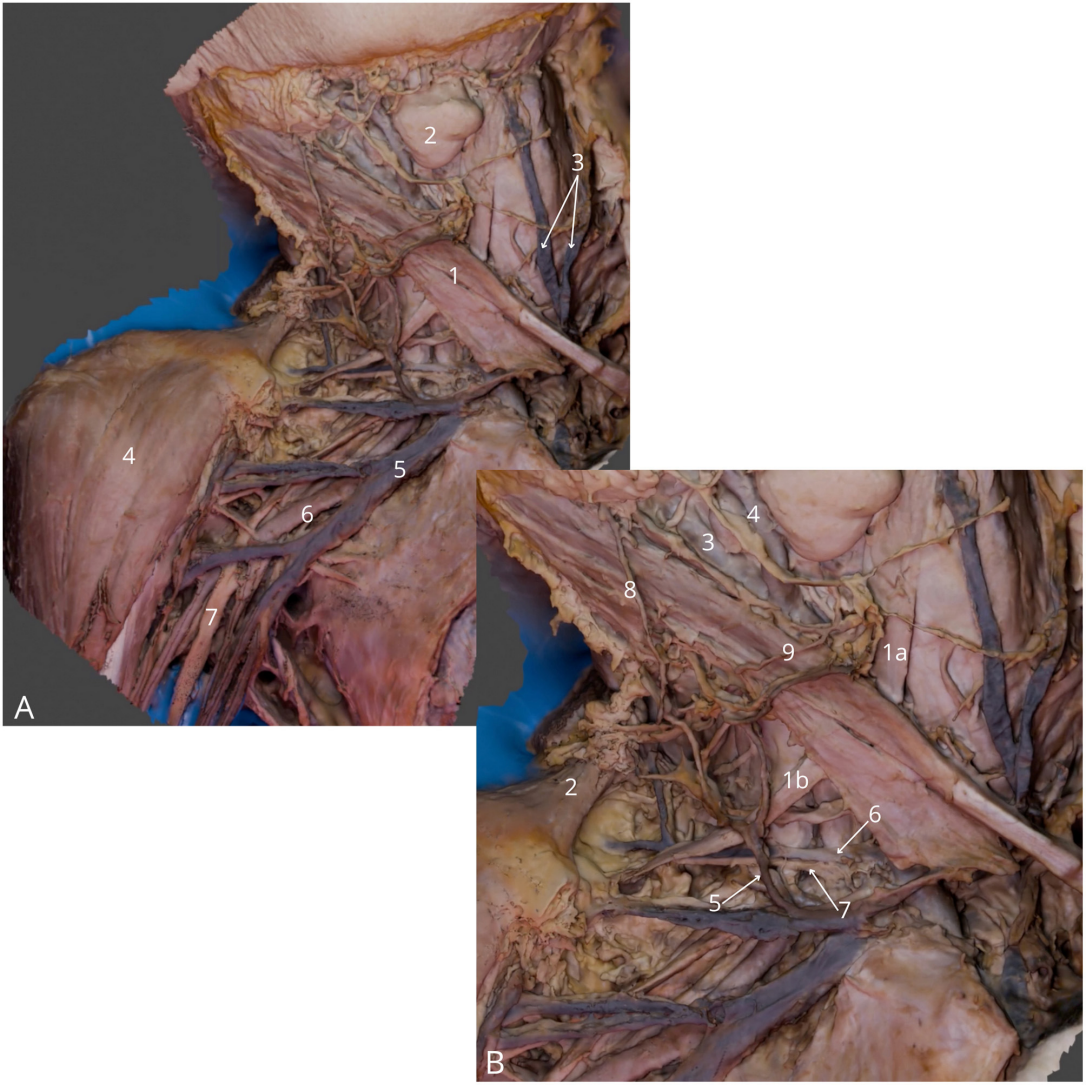
Neck and axillary fossa dissection. **(A)** General view, highlighting right sternocleidomastoid muscle (1), right submandibular gland (2), anterior jugular veins (3), right deltoid muscle (4), right axillary vein (5), right axillary artery (6), right median nerve (7); **(B)** Detailed view at the lateral triangle of the neck, highlighting right omohyoid muscle—superior (1a) and inferior (1b) belly, right trapezius muscle (2), right internal carotid artery (3), right external carotid artery (4), right external jugular vein (5), right transverse cervical vein (6), right transverse cervical artery (7), right great auricular nerve (8), right transverse cervical nerve (9). The 3D model of this specimen is available in the supplementary material as Supplementary Video S4.

**Figure 5:**
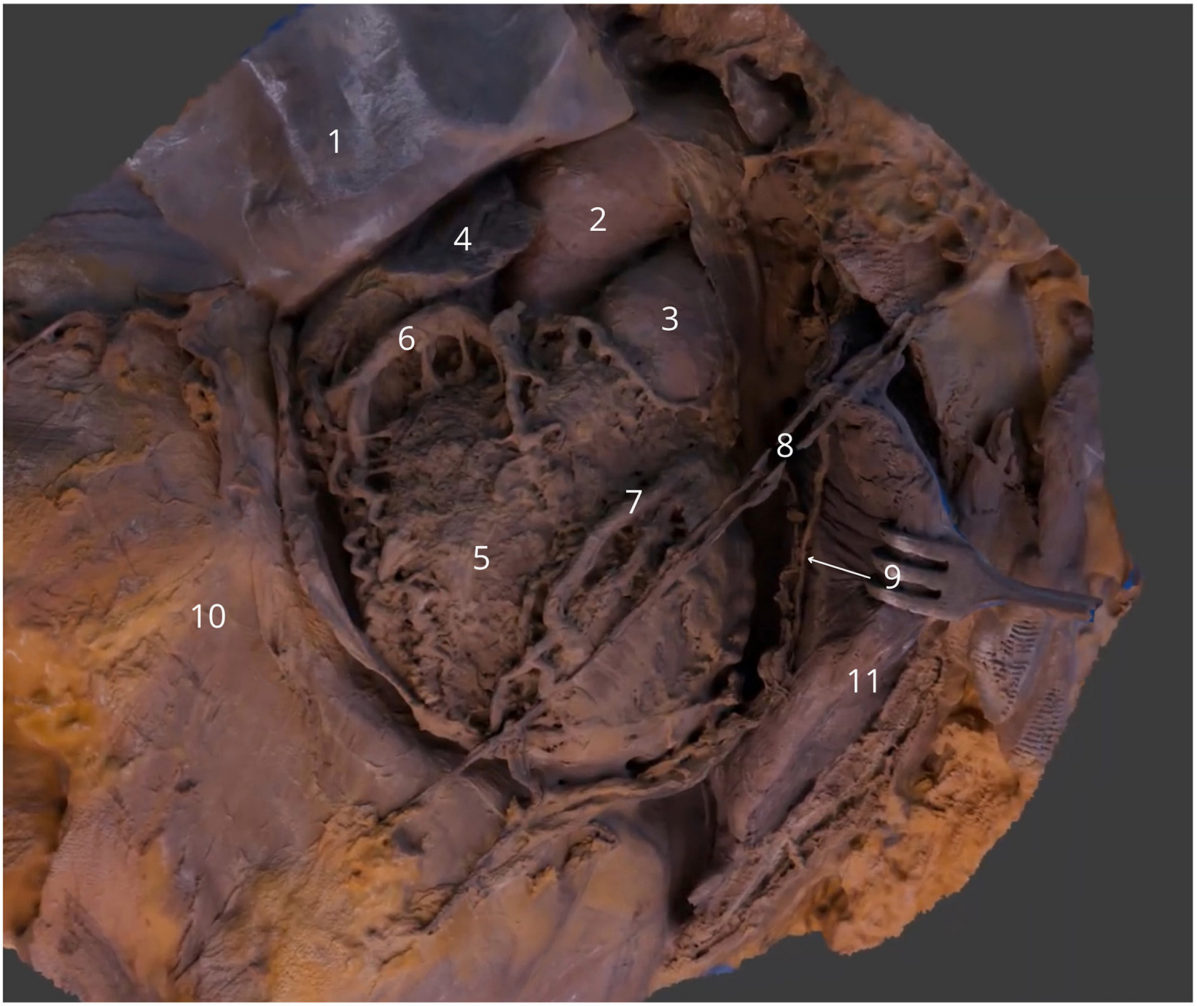
Sternocostal surface of the heart in the thoracic cavity. Lifted pericardial sac (1) with highlighted ascending aorta (2), pulmonary trunk (3), right appendage (4), right ventricle (5), right coronary artery (6) and anterior interventricular branch of left coronary artery (7). Left internal thoracic vessels (8), left phrenic nerve (9), diaphragm (10) and left lung (11). The 3D model of this specimen is available in the supplementary material as Supplementary Video S5.

**Figure 6:**
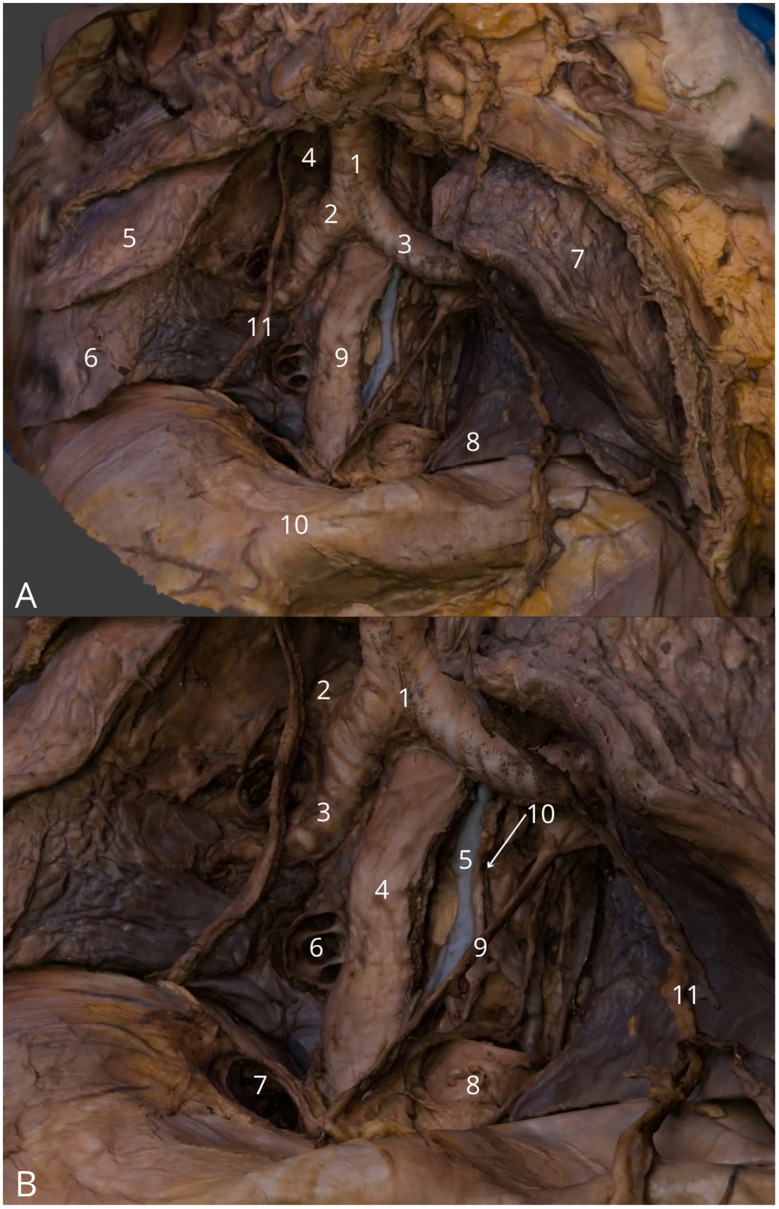
Posterior mediastinum. **(A)** General view highlighting trachea (1), right main bronchus (2) and left main bronchus (3), superior vena cava - cut (4), superior lobe of the right lung (5), inferior lobe of the right lung (6), superior lobe of the left lung (7), lingula of the lung (8), esophagus (9) and diaphragm (10) with right phrenic nerve (11); **(B)** Detailed view on tracheal bifurcation (1), eparterial bronchus (2), hyparterial bronchus (3), esophagus (4), hemiazygos vein (5), right inferior pulmonary vein (6), caval opening (7), thoracic aorta – cut (8), left vagus nerve (9), left recurrent laryngeal nerve (10) and left phrenic nerve (11). The 3D model of this specimen is available in the supplementary material as Supplementary Video S6.

**Figure 7:**
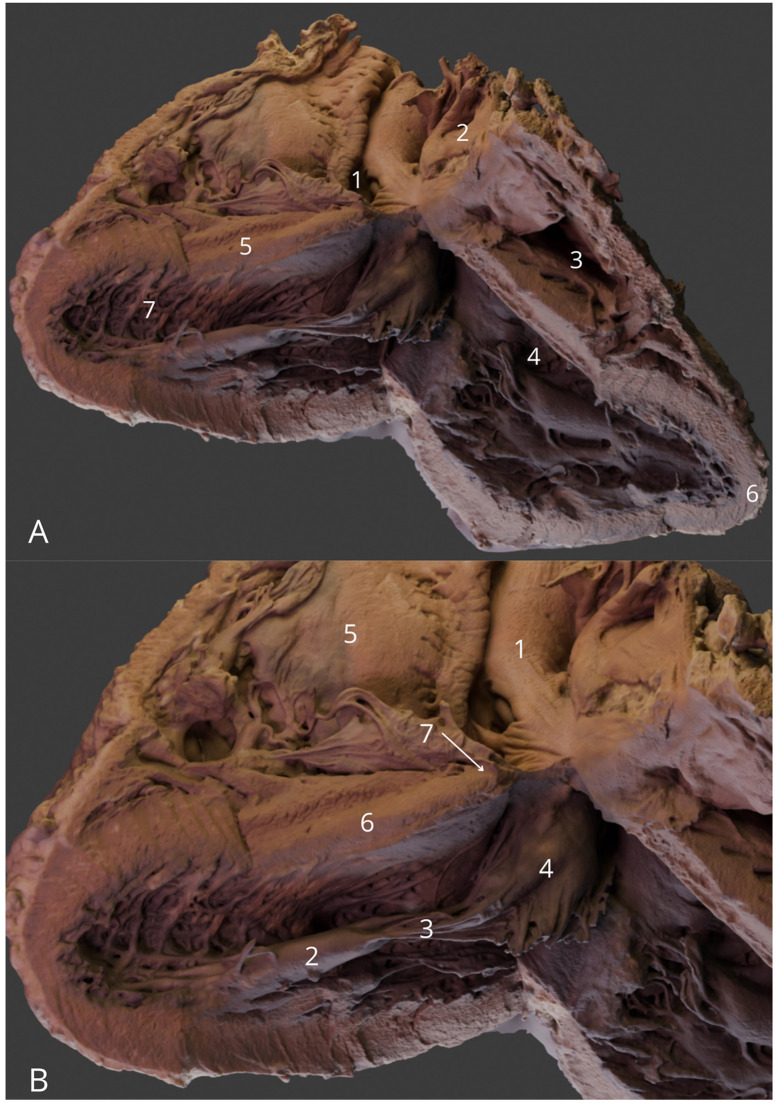
Heart—four chamber view **(A)** General view: right atrium (1), left atrium (2), right ventricle (3), left ventricle (4), interventricular septum (5), apex of the heart (6) and trabeculae carneae (7); (**B)** Focus on the internal structure of the ventricles with ascending aorta (1), left anterior papillary muscle (2), chordae tendineae (3), mitral valve leaflet (4), tricuspid valve leaflet (5), interventricular septum with muscular part (6), and membranous part (7). The 3D model of this specimen is available in the supplementary material as Supplementary Video S7.

**Figure 8:**
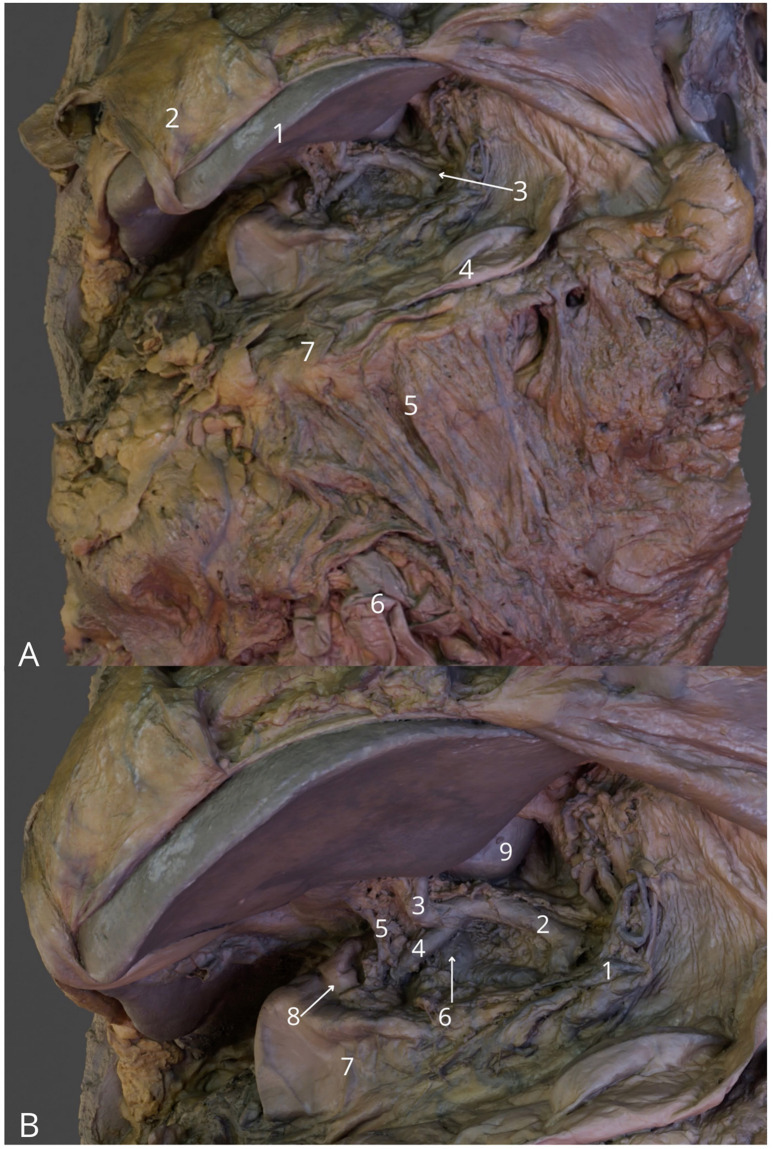
Abdominal cavity, anterior view. **(A)** General view with liver (1), falciform ligament of the liver (2), celiac trunk (3), greater curvature of stomach (4), epiploe (5), gastrocolic ligament (7); **(B)** Celiac trunk and its branches: left gastric artery (1) common hepatic artery (2) proper hepatic artery (3) gastroduodenal artery (4). Bile duct (5), portal vein (6), pylorus of stomach (7), bulb of duodenum (8), quadrate lobe of the liver (9). The 3D model of this specimen is available in the supplementary material as Supplementary Video S8.

**Figure 9:**
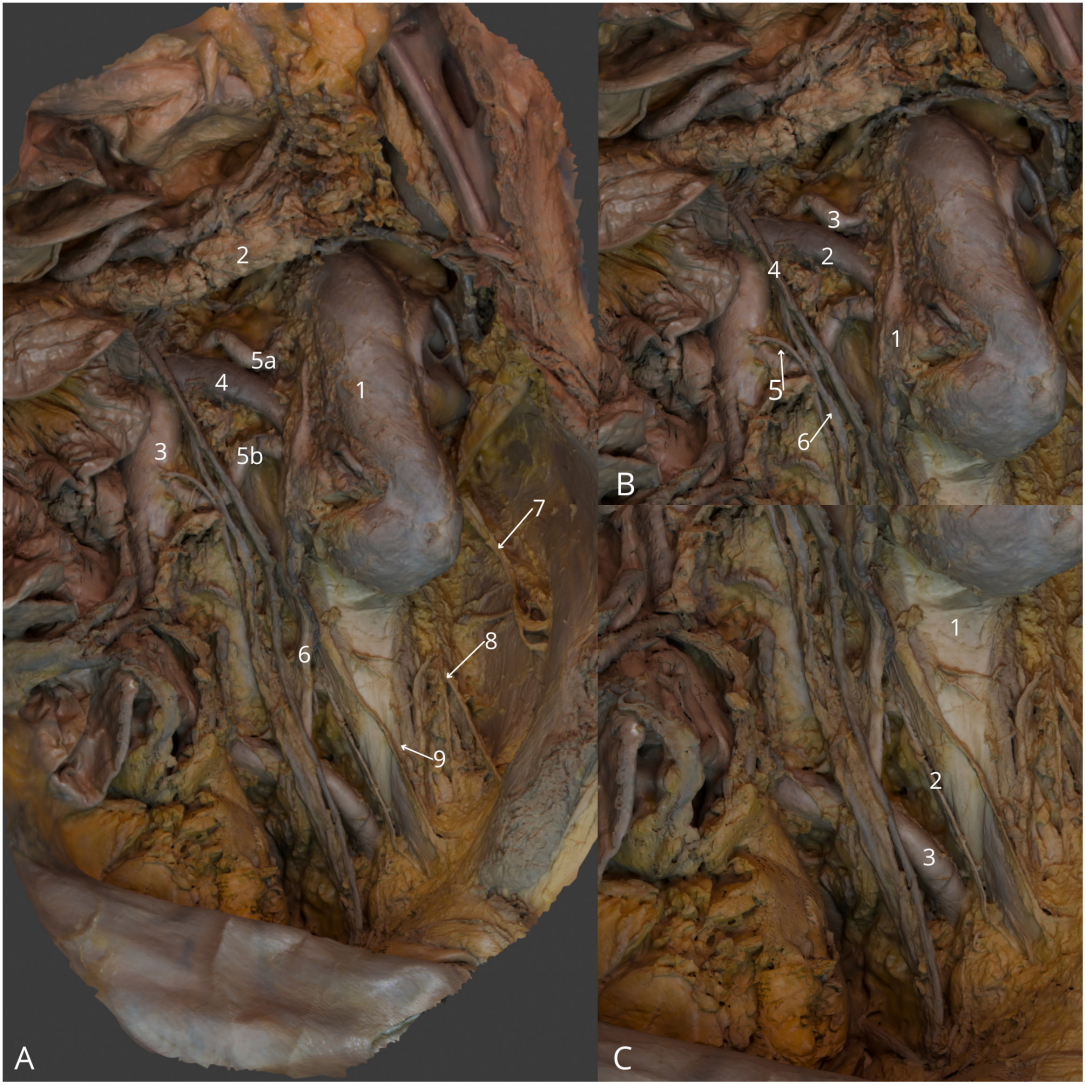
Left kidney in retroperitoneal space, anterior view. **(A)** General view: left kidney (1), body of pancreas (2), abdominal aorta (3), left renal vein (4), double left renal artery (5a, 5b), left ureter (6), left iliohypogastric nerve (7), left ilioinguinal nerve (8), and left lateral cutaneous nerve of thigh (9); **(B)** Zoom at left kidney with left ureter (1), left renal vein (2), left superior renal artery (3), inferior mesenteric vein (4), inferior mesenteric artery (5), and left testicular vein (6); **(C)** Zoom at the left inferior quadrant with marked left psoas major muscle (1), left genitofemoral nerve (2), and left common iliac artery (3). The 3D model of this specimen is available in the supplementary material as Supplementary Video S9.

**Figure 10:**
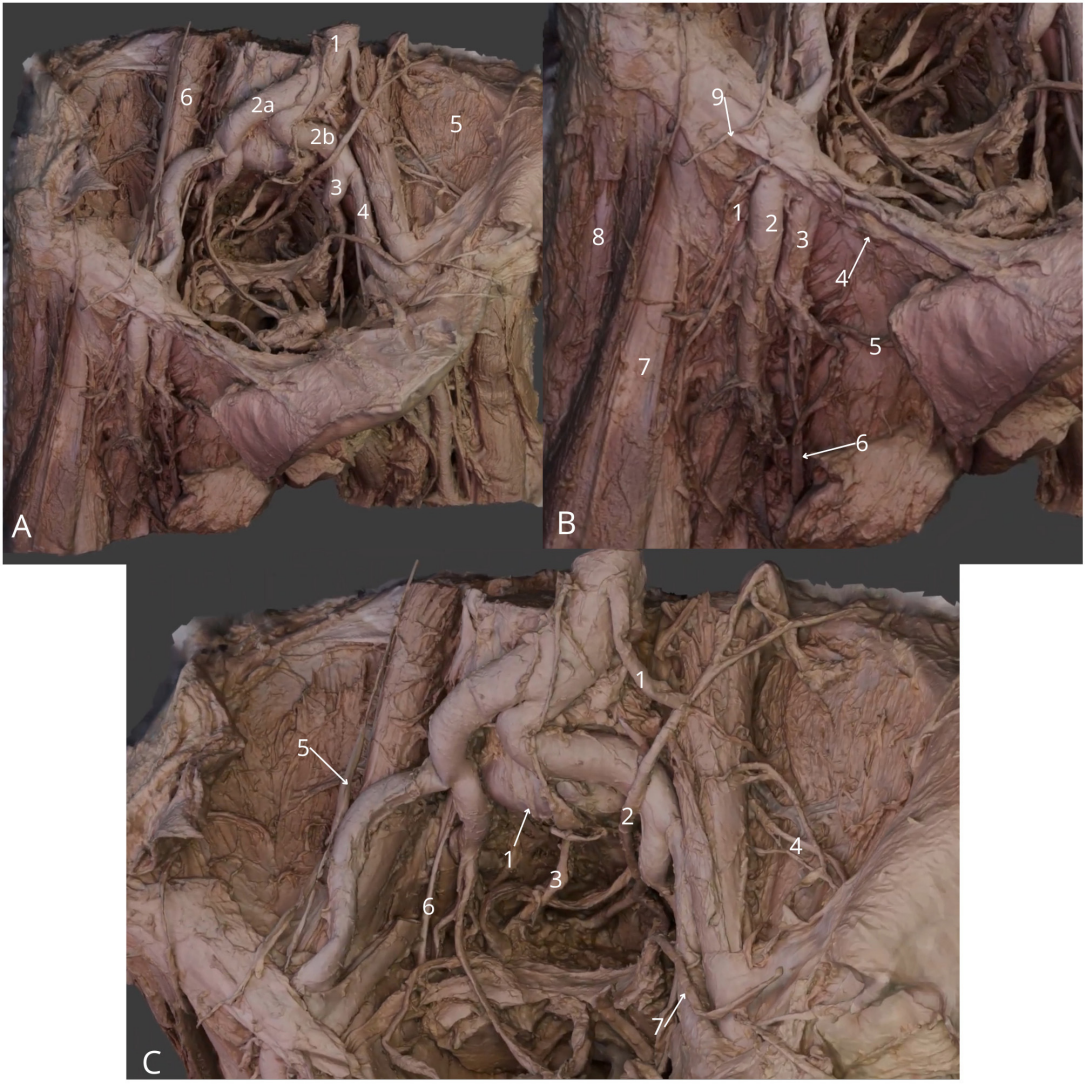
Male pelvic cavity dissection, femoral triangle region. **(A)** General view: abdominal aorta (1), right common iliac artery (2a), left common iliac artery (2b), left internal iliac artery (3), left external iliac artery (4), left iliacus muscle (5) and left psoas major muscle (6); (**B)** Detailed view at femoral triangle: right femoral nerve (1), right femoral artery (2), right femoral vein (3), right inguinal ligament (4), right external pudendal vein (5), right great saphenous vein (6), right sartorius muscle (7), right tensor fasciae latae muscle (8) and right lateral cutaneous nerve of thigh (9); **(C)** Zoom at the pelvic cavity: median sacral artery (1), left ureter (2), right hypogastric nerve (3), left lateral cutaneous nerve of thigh (4), right genitofemoral nerve (5), right obturator nerve (6), left vas deferens (7). The 3D model of this specimen is available in the supplementary material as Supplementary Video S10.

**Figure 11:**
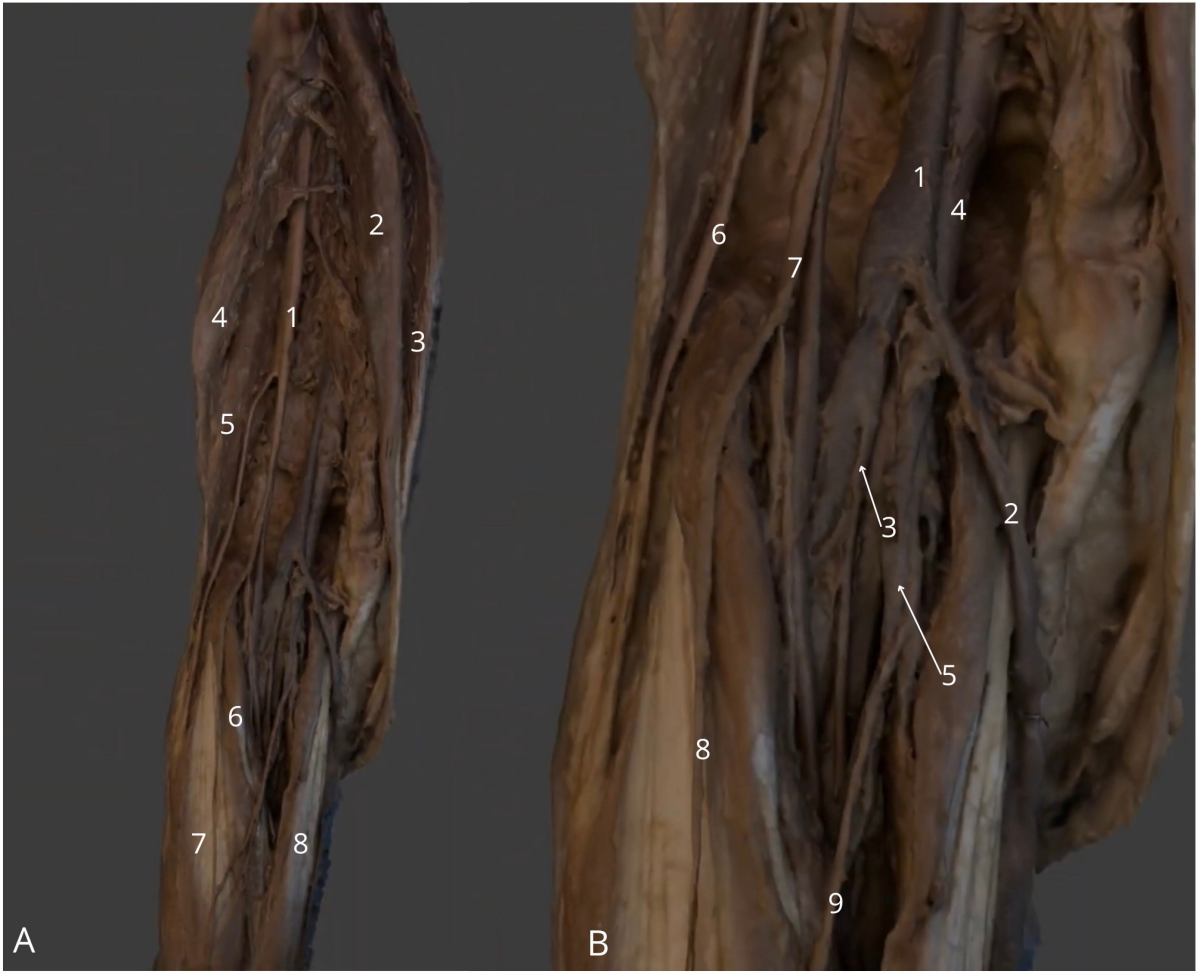
Left lower limb, posterior side with popliteal fossa. **(A)** General view: left sciatic nerve (1), left semitendinosus muscle (2), left semimembranosus muscle (3), long head of left biceps femoris (4), short head of left biceps femoris muscle (5), left plantaris muscle (6), left gastrocnemius muscle - lateral head (7) and medial head (8); **(B)** Zoom at popliteal fossa with visible left popliteal vein (1), left small saphenous vein (2), left posterior tibial vein (3), left popliteal artery (4), left posterior tibial artery (5), left common fibular nerve (6), left tibial nerve (7), left lateral cutaneous sural nerve (8) and left medial cutaneous sural nerve (9). The 3D model of this specimen is available in the supplementary material as Supplementary Video S11.

Table 2 outlines the models, descriptions, dissection details, and corresponding figures and videos. The videos of every specimen are available in the Supplementary materials.

**Table 2:** List of 3D models, highlighting the key dissection details, with corresponding figures (Figures 1–11) and videos (Supplementary Videos S1–S11).

**Model number**	**Model description**	Specimen number^*^	**Dissection details**	**Corresponding figure**	**Corresponding video**
1	Cerebrum and spinal cord	1	Arachnoid mater removed on the right hemisphere	Figures 1A–D	Supplementary Video S1
2	Intracranial structures	2	Right cerebral hemisphere, right orbital roof and right tympanic tegmentum removed, the right part of tentorium cerebelli lifted	Figures 2A, B	Supplementary Video S2
3	Region of the face	3	Skin removed only in areas surrounding the course of the highlighted and colored vessels and nerves (arteries highlighted in red, veins highlighted in blue, and nerves highlighted in yellow)	Figures 3A, B	Supplementary Video S3
4	Neck and axillary fossa dissection	4	Right platysma removed, corpus of the right clavicle and sternum cut out	Figures 4A, B	Supplementary Video S4
5	Sternocostal surface of the heart in the thoracic cavity	4	Anterior thoracic wall removed, pericardial sac lifted, left lung retracted to the left with a retractor	Figure 5	Supplementary Video S5
6	Posterior mediastinum	4	Heart within the pericardial sac, pretracheal vessels and thoracic aorta with its branches removed	Figures 6A, B	Supplementary Video S6
7	Heart—four-chamber view	5	Cut longitudinally along the axis of the heart on the anterior wall, exposing both atria and ventricles	Figures 7A, B	Supplementary Video S7
8	Abdominal cavity, anterior view	6	Anterior abdominal wall and the lesser omentum removed, liver with the round ligament and falciform ligament lifted cranially and to the right	Figures 8A, B	Supplementary Video S8
9	Left kidney in retroperitoneal space, anterior view	6	The small intestine with transverse, descending and sigmoid colon lifted outwards and to the right, the left lateral wall of the abdomen was cut longitudinally along the middle axillary line	Figures 9A–C	Supplementary Video S9
10	Pelvic cavity dissection with femoral triangle regions	7	Cadaveric corpus cut horizontally at the level of L5 vertebral corpus, and horizontally through both thighs; anterior abdominal wall lifted anteriorly and to the left with other abdominal walls removed; urinary bladder, uterus and rectum removed from pelvic cavity	Figures 10A–C	Supplementary Video S10
11	Left lower limb, posterior side with popliteal fossa	8	Hamstrings muscles retracted outwards	Figures 11A, B	Supplementary Video S11

^*^All dissected specimens that were scanned, are listed in Table 1. The videos of every specimen are available in the Supplementary materials.

## 4 Discussion

### 4.1 Technological advancements in clinical anatomical education

This study demonstrates a valuable learning tool using cutting-edge 3D digital technology for anatomical education and professional medical practice. It offers the opportunity to develop a deep understanding of the complex topographical organization of anatomical features, which is key for further analysis of pathological processes (11, 13, 14). There has been considerable debate among academics and medical trainees not about the quantity of anatomy taught, but about the relevance and effectiveness of teaching methods employed (15, 16). Studying the relationship between anatomical education and professional practice is intricate and demanding due to the wide variation in individual experiences and the difficulty of clearly defining knowledge and its application in professional settings. In the cognitive process, an important role is played by the gradual and smooth transition through subsequent stages of knowledge combined with their integration and building on additional information (11, 13, 17). As a result, in medical practice, it is important to revisit the basic sciences including anatomical foundations (11, 13, 14).

Current readily accessible study resources mostly offer 2D representations typically found in textbooks or atlases, which often fall short in conveying the intricacies of the multiple planes and spatial relationships, thereby limiting understanding (18). Anatomy is fundamentally a three-dimensional subject (10), and the benefits of three-dimensional learning tools are now undeniable and widely recognized (19). In view of this study, integrating 3D visualization technology with traditional cadaveric method of teaching, has the potential to improve spatial cognition, refine surgical techniques, and makes high-fidelity training more accessible. Multiple studies have demonstrated that volumetric visualization enhances learner's ability to identify and localize anatomical structures (9, 19, 20). 3D images offer an innovative approach, enhancing both student education and clinical training of novice trainees especially in the target stage—operation planning, which can be seen in the medical fields such as neuroanatomy, abdominal surgery, tumor anatomy, cardiology, rheumatology, immunology and many others (2, 21–23).

While other techniques exist for creating 3D models, including 3D segmentation from magnetic resonance (MR) or computed tomography (CT), pre-acquired images (12, 24, 25), structured-light, surface 3D scanning offers the advantage of capturing more realistic features, colors, and textures of the specimen of interest in more efficient and accessible workflow. Another advanced 3D visualization method, photogrammetry, is based on overlapping two-dimensional photographs taken from different angles and converting them into 3D digital models (26, 27). The quality of photogrammetric 3D models, strongly depends on the resolution of the photographs taken, requiring expensive setup of multiple cameras and sophisticated software for 3D reconstruction (12, 27).

The 3D scanner used in this study stands out amongst other methods for its high precision, portable handheld design, allowing easy visualization of versatile objects. According to certain studies, this method of 3D scanning provides more accurate registration of anatomical structures, and the obtained images exhibit less geometric distortion (12, 28). The 3D scanner can be utilized in remote locations or without power supply (with the attached battery pack). The scanning process can be possible after plugging the Artec 3D Space Spider scanner to a computer with installed Artec Studio Professional software. The scanner's technology uses hybrid geometry and color tracking technologies for the highest quality data acquisition and faster processing. This means no targets are required to achieve accurate results.

While several prior studies have successfully demonstrated 3D scanning and photogrammetry-based reconstructions of cadaveric specimens, our study introduces distinct elements that set it apart in terms of workflow, fidelity, and applicability. Notably, previous works from Barrow, Miami, and Yeditepe Universities have produced highly impactful contributions in the field, such as developing simplified photogrammetry workflows for cadaveric specimens (29), generating detailed augmented and virtual reality (VR) simulations of cerebral white matter anatomy (30), and producing extended-reality fiber dissection models of the cerebellum and brainstem (31). These studies underscore the value of AR/VR-enhanced models for neurosurgical education and research.

In contrast, our methodology focuses on high-fidelity surface 3D scanning of formalin-fixed cadavers, which are more commonly available in standard anatomical laboratories worldwide, rather than relying exclusively on fresh specimens or highly specialized photogrammetry setups. By using a structured-light handheld scanner, we achieved accurate surface texture capture without requiring multiple camera arrays or extensive photogrammetry calibration, thereby offering a more accessible and efficient workflow for widespread adoption. Furthermore, our integration of 3D reconstructions with corresponding 2D cadaveric dissection images provides a dual-format learning tool that enhances both spatial understanding and structural recognition. This hybrid approach is relatively underexplored in the existing literature and offers a novel pedagogical advantage.

By situating our work alongside these pioneering efforts, we highlight its unique contribution: a scalable and accessible protocol for generating high-resolution 3D anatomical models from formalin-fixed specimens, complemented by integrated 2D references, making it well-suited for both anatomy education and clinical training. The contribution to the broad dissemination of anatomical knowledge with the presented method, can be achieved by hosting acquired 3D models on digital platforms, allowing users to access them on personal devices such as computers or mobile phones at no additional cost. Moreover, the creation of digital libraries of anatomical specimens allows documentation of anatomical variations that may be otherwise difficult to identify routinely in various laboratory settings. Therefore, students, educators, and medical professionals can gain easy access to such invaluable resources without financial constraints unlike other methods, such as cadaveric dissection or anatomical literature.

Another method, particularly gaining attraction recently, is the use of virtual reality (VR) headsets. Despite the advantage of viewing real-life 3D images integrated into reality such devices require costly VR equipment, regular technical maintenance and the physical discomfort experienced by users must all be taken in account. Studies have demonstrated that VR headset induce symptoms aligning with cybersickness, such as nausea, head pain, visual discomfort, and disorientation (32, 33), limiting the time in which the technology can be comfortably used, reducing the effectiveness of VR as a learning tool (34, 35). Nevertheless, 3D models as produced in this study, could be uploaded to such headsets which would supplement the learners experience by the real-life cadaveric images.

Additionally, apposing 2D images and interactive 3D models—as implemented in this study—presents a novel approach that accentuates the advantages of both imaging formats and provides a comprehensive platform for grasping the anatomical complexity. This unique combination of imaging not only has the potential to enrich an interactive learning experience, but enhance clarity in identifying anatomical landmarks, establishing a robust foundation for advanced clinical interpretations.

Notably, using a visually engaging and interactive learning tool like the method introduced in this study, can potentially reduce the cognitive load on the learner, and thus facilitate more effective learning by stimulating the repetition stage, necessary for the transition of information from working memory to long-term memory (11, 17). This is noted to be crucial aspect in freeing up limited working memory, which can lead to integrating many areas of knowledge and skills at the same time (13). The goal of this process is to develop three-dimensional image into a mental model within the physician's mind, aiding clinical practice (11).

### 4.2 Integrating virtual surgical simulation and digital twin technologies

While our study primarily focuses on anatomical education using handheld 3D scanning, recent advances in virtual surgical simulation and digital twin technologies warrant further discussion. Such developments have significant implications for enhancing training and real-time surgical rehearsal, where precise anatomical visualization is essential.

Several studies demonstrated advantages of application of augmented reality (AR) and digital twin models for preoperative planning and intraoperative guidance in various surgical fields (36, 37). Digital twins are useful for real-time simulations that replicate patient-specific anatomy and pathology, supporting precision medicine and improving surgical outcomes (38). Visualizing small vessels, particularly if the cadaver is not fresh, presents significant challenges in terms of quality of image and reproducibility, hence the use of contrast enhanced imaging (39), initially validated for purposes of virtual surgery (40, 41), has been proposed to create digital twins for medical education with encouraging initial results (42). Virtual reality (VR) based microsurgical training models, particularly in neurosurgery, have also evolved significantly, for example, tools like the NeuroTouch simulator have proven useful for refining skills in microvascular anastomosis, aneurysm clipping, and tumor resection (36, 43).

These innovative technologies demonstrate how reproducible, high-fidelity virtual environments can improve procedural accuracy. Importantly, many existing platforms have achieved reproducibility and transferability across specialties such as otolaryngology, urology, and cardiothoracic surgery, validating the scalability of such virtual training models (44, 45).

We hope that in the future, there will be many opportunities for integration of our cadaver-derived 3D models into such AR/VR/digital twins platforms, enabling hybrid systems that combine real anatomical data with dynamic simulation technologies. By incorporating and comparing our models with these virtual platforms, we envisage a next-generation educational platform; one that merges real anatomical accuracy, clinical context, and procedural feedback. This would provide a compelling and educationally rich environment for students and trainees across a variety of surgical disciplines.

### 4.3 New perspective on the cadaver-based study

The study of anatomy is inextricably linked to the use of human cadavers. Dissection offers material abundant in anatomical variations (14, 46) giving opportunity for better understanding the differences and individualities occurring in particular cases.

Despite the clear advantages of cadaveric dissections, there are several disadvantages that underscore the need for more modern solutions in anatomical education. These include low and unequal availability of cadavers, expensive preservation regimen, time-consuming specimen preparation, potential health hazards, and for some, religious moral concepts and inability to restore damaged structures (6).

A significant debate has emerged regarding which chemicals are best to use in terms of cadaver preservation to prevent fast tissue degradation. Some non-formalin-based alternatives include ethanol-glycerin, pickling salts and a patented Bronopol solution (47). However, the widespread use of formalin-based solution, containing formaldehyde as the fixing agent diffused in alcohol is still commonly used in facilities, despite the risks of formaldehyde being acknowledged and proven as carcinogenic and mutagenic to humans (1, 5). Therefore, the need for a formalin-free and fresh-like cadaver in cadaver-based education. Some preservation alternatives have been explored but are incomparable to results provided by formalin use (47, 52). In terms accessibility of dissecting rooms, doctors lack access to these spaces once they have completed their medical studies and begun clinical work. Nevertheless, online resources parenting human cadaveric images are quite uncommon or unknown to many clinicians.

This research presents a solution: a virtual platform with models accurately representing anatomical structures from various donors providing imperative benefits in standard clinical practice. Moreover, 3D models in a digital format are sustainable, overcoming the inevitable issue of specimen degradation and dependency on body donation programmes.

### 4.4 Limitations

Despite the advantages of the cadaveric dissections and the use of 3D imaging technology, there are some limitations to the presented method in this research. The scanner used in this study is designed for visible light usage, therefore the quality of scans depends on numerous factors such as lighting conditions, tissue's light reflectivity and hydration levels, as well as the background for scanning and the position of the device and specimen. Not meeting these conditions can result in the low-quality images, making difficult to create real-life reconstructions. Also, the scanner's resolution of 0.1 mm restricted visualizing texture details smaller than 0.1 mm. Similarly, the ability to capture depth and spatial relationship between some parts located at different depth levels proved difficult. To address these imperfections, some adjustments on the Blender^®^ software were used, to effectively enhance the image quality and resolution. However, it is worth noting that this solution demanded a nuanced understanding of the software's settings, underscoring the importance of technical proficiency when working with intricate 3D imaging technology.

Another limitation is that although the presented models are described as valuable educational tools, no direct validation with students or trainees was conducted in this study. While this was not the primary aim, future research should focus on pedagogical validation to assess the effectiveness of such models in anatomy education. Previous studies have already demonstrated that 3D models, augmented reality, and virtual reality applications can significantly enhance learning outcomes, spatial understanding, and engagement in medical education (48–51).

Despite these limitations, the integration of dissection and 3D models in anatomy education opens new horizons for understanding complex spatial anatomical relations. These tools offer clinicians and students engaging, interactive, and readily accessible learning experiences, complementing traditional 2D resources, in a format accessible to all.

## 5 Conclusion

Interactive 3D models of human cadavers allow to bridge the gap between practical application and theoretical knowledge eliminating challenges that come with practicing on cadavers. In addition to fast, easy and widespread access, the low maintenance costs make this technology a strong contender in the educational and clinical sector. Integrating advanced technologies and reliable study materials promises to strengthen anatomical fluency and improve clinical outcomes.

## Data Availability

The original contributions presented in the study are included in the article/Supplementary material, further inquiries can be directed to the corresponding author.
